# Prognostic value of hemoglobin A1c in nondiabetic and diabetic patients with acute ischemic stroke

**Published:** 2016-10-07

**Authors:** Mohammad Ali Shafa, Hosseinali Ebrahimi, Farhad Iranmanesh, Mojtaba Sasaie

**Affiliations:** 1 Department of Neurology, Afzalipour School of Medicine, Neurology Research Center, Kerman University of Medical Sciences, Kerman, Iran; 2 Department of Neurology, Afzalipour School of Medicine, Kerman University of Medical Sciences, Kerman, Iran

**Keywords:** Hemoglobin A1c, Stroke, Prognosis

## Abstract

**Background:** Diabetes is a well-known risk factor for acute ischemic stroke (AIS). Some recent studies point to hemoglobin A1c (HbA1c) may have prognostic value in nondiabetic and diabetic patients with ischemic stroke (IS). The aim of this study was to evaluate the prognostic value of HbA1c on mortality and morbidity in AIS patients with and without diabetic.

**Methods:** In this prospective observational study, 150 diabetic and nondiabetic patients with AIS were evaluated for serum HbA1c level, hypertension (HTN), hyperlipidemia, and smoking in the first 24 hours of admission to determine their value to predict mortality and mortality at 30 and 90 days. Morbidity was estimated by the National Institutes of Health Stroke Scale (NIHSS) and follow-up visits were scheduled 30 and 90 days after admission. Results were analyzed with independent t-test and logistic regression analysis.

**Results:** In this study, 73 patients (48.7%) were female and the rest were men. At 30 days, the diabetic patients had a significantly higher mortality, but no significant difference was found between diabetics and morbidity. No significant statistical differences were seen between HbA1c and 30 and 90 days with mortality and morbidity among diabetic patients. Furthermore, no significant statistical difference was seen between HbA1c and 30 and 90 days morbidity and between HbA1c and 30 days mortality in nondiabetic patients. However, in nondiabetic patients, on multiple logistic regression analysis, a significant correlation was seen between 90 days month mortality and HbA1c (P = 0.002).

**Conclusion:** HbA1c can be as a predictive biomarker in nondiabetic patients with AIS.

## Introduction

Stroke is one of the most important causes of death in the world. It is the second cause of mortality in many countries. The frequency of acute ischemic stroke (AIS) is about 75%.^1^ Diabetes are one of the main risk factors for AIS^2^ and has repeatedly shown a negative effect of exacerbating ischemic brain injury, accelerating the molecular processes leading to cell death, and resulting finally in larger infarct volumes and poorer outcomes.^3^^,^^4^ It seems that the effect of diabetes on stroke is mainly due to atherosclerosis or induction of an inflammatory reaction which is possibly reversible with treatment.^4^^,^^5^ Some studies show that some blood sugar related biomarkers, such as fasting blood sugar or random blood sugar, can be used as prognostic tools in ischemic stroke (IS) patients.^6^^-^^9^ Hemoglobin A1c (HbA1c) is the form of Hb. It is measured to estimate the average plasma glucose concentration over 2-3-month period. Some recent studies suggest that HbA1c may have prognostic value in patients with AIS.^10^ For example, in an Irish study, in 165 patients who underwent surgery for vascular disorders, the rate of 1-month mortality was significantly higher in nondiabetic and diabetic patients with abnormal HbA1c level.^9^ Another study on diabetic and nondiabetic patients with abnormal HbA1c level showed higher carotid artery stenosis in patients with abnormal HbA1c.^11^ In other studies in China on patients with AIS, the severity of stroke was higher in patients with abnormal HbA1c.^12^^,^^13^ Despite above-mentioned studies, a study in Korea on diabetic patients showed a correlation between HbA1c level and severity of stroke, but this relationship was not seen in nondiabetic patients.^14^ According to these controversies and lack of a final conclusion, this study aimed to evaluate serum HbA1c level and its prognostic value in nondiabetic and diabetic patients with IS.

## Materials and Methods

Our prospective observational study was performed on 150 patients with AIS in Kerman. Patients suffered from IS for the first time and were admitted within first 24 hours of onset of symptoms. Diagnosis of IS was based on computed tomography-scan and magnetic resonance imaging (DWI, T1, and T2) findings. A cardiologist visited all patients, and the patients underwent transthoracic echocardiography and electrocardiography monitoring for 24 hours. In the case of clinical suspicion, transesophageal echo was done and cardioembolic stroke was excluded. The patients with any underlying diseases other than diabetes and hypertension (HTN) were excluded from the study. Those who took medications (except drugs for diabetes, HTN, and hyperlipidemia) were also excluded. Furthermore, patients with any laboratory abnormalities such as elevated erythrocyte sedimentation rate were excluded from our study. In this study, those patients undergoing drug therapy or having systolic blood pressure higher than 140 or diastolic higher than 90 mmHg were considered as having HTN. Patients were also treated diabetic who were under drug therapy or had fasting blood glucose > 126 mg/ml or random blood sugar over 200 mg/dl with the symptoms of diabetes. Those who used five cigarettes/day were regarded as smokers. HbA1c was measured within the first 24-hour of admission by chromatography and HbA1c level > 6.5 ug/ml was considered as abnormal in nondiabetic patients. In addition, HbA1c level < 7 ml was regarded as good control (normal) in diabetic patients.^15^^,^^16^ The National Institutes of Health Stroke Scale (NIHSS) score was assessed on admission and 30 and 90 days to get morbidity assessment. The patients fallowed for 90 days or death. For every patient, a questionnaire containing demographic information and NIHSS was provided, and patients were divided into two groups according to diabetes. Power of study was 80% and P ≤ 0.050 was considered statistically significant. Demographic information and other findings were analyzed using independent t-test and logistic regression. Our study was approved by the Ethics Committee of Kerman University of Medical Sciences.

## Results

In this study, 73 (48.7%) and 77 (51.3%) patients were female and male, respectively. Table 1 shows baseline characteristics data of the patients. The mean HbA1c in diabetic and nondiabetic patients were 7.72 ± 2.20 and 5.68 ± 1.22, respectively. 

**Table 1:** Baseline characteristics data of the patients

**Characteristics**	**Value**		
Age (year) (mean ± SD)		71.18 ± 9.10	
Gender [n (%)]			
Female	73 (48.7)		
Male	77 (51.3)		
History of HTN [n (%)]			
Yes	100 (66.7)		
No	50 (33.3)		
History of diabetes [n (%)]			
Yes	46 (30.7)		
No	104 (69.3)		
History of smoking [n (%)]			
Yes	56 (73.3)		
No	94 (62.7)		
History of hyperlipidemia [n (%)]			
Yes	57 (38.0)		
No	93 (62.0)		
Level of HbA1c (mean ± SD)	6.31 ± 1.84		
30 days mortality [n (%)]	32 (21.3)		
90 days mortality [n (%)]	8 (5.3)		

SD: Standard deviation; HTN: Hypertension; HbA1c: Hemoglobin A1c

**Table 2:** Comparison of morbidity among diabetic and nondiabetic patients

**Time**	**Diabetic patients (mean ± SD)**	**Nondiabetic patients (mean ± SD)**	**P**
Admission time morbidity	10.42 ± 1.09	8.37 ± 0.55	0.101
30 days morbidity	5.86 ± 0.72	5.53 ± 0.57	0.753
90 days morbidity	5.55 ± 0.76	4.79 ± 5.03	0.472

SD: Standard deviation

At 30 days, patients with diabetic had a significantly higher mortality (P = 0.040), but there was no significant difference between diabetes and morbidity (Tables 2 and 3). There was no significant statistical difference between HbA1c and 30, and 90 days with mortality and morbidity among diabetic patients. Furthermore, there was no significant statistical difference between HbA1c and 30, and 90 days morbidity and between HbA1c and 30 days mortality in nondiabetic patients. However, in nondiabetic patients, on multiple logistic regression analysis, a significant correlation was seen between 90 days month mortality and HbA1c (P = 0.002) (Table 4). Regarding insignificant difference in NIHSS on admission in dead and alive nondiabetic patients (P = 0.890), it can be concluded that HbA1c is a prognostic biomarker in nondiabetic patients.

## Discussion

In stroke, reliable prognostic markers are very important because they can aid clinical decision-making and help to health-care resources. The aim of our study was to evaluate the prognostic value of HbA1c in non-diabetic and diabetic patients with AIS. Our findings showed no significant difference in HbA1c level on admission with 30 and 90 days mortality and morbidity in diabetic patients, and with 30 and 90 days morbidity and 30 days mortality in nondiabetic patients. However, our findings showed a significant correlation between 90 days mortality in non-diabetic patients and HbA1c after adjustment for risk factors by logistic regression analysis (P = 0.002). Then, it can be concluded that HbA1c can be used as a prognostic biomarker in nondiabetic patients with AIS. This result was agreement with some previous studies. For example, Roquer et al.^16^ aimed to evaluate the effect of HbA1c and glucose level on 3-month mortality predilection in nondiabetic and diabetic IS patients. They found HbA1c determination combined with first measured glucose value is useful to stratify mortality risk in AIS patients.^16^ Hjalmarsson et al.^17^ in a retrospective study on 501 patients with AIS observed a correlation between HbA1c and mortality and also permanent complications in nondiabetic patients, as same as diabetic patients. Guo et al.^13^ in another study evaluated HbA1c in 180 patients IS within the first 24-hour of stroke. They divided the patients into three groups based on HbA1c level (6.5 > HbA1c > 5.7, HbA1c < 5.5, and HbA1c < 6.5). They evaluated them at the admission time and 3-month after stroke by NIHSS. Their study showed that IS prognosis depends on different HbA1c levels. The patients with higher HbA1c level had poorer neurological condition and prognosis in the first 3 months after stroke.^13^ Some studies show that HbA1c was associated with greater carotid stenosis and periventricular ischemic lesions.^18^^-^^20^ Studies even suggest that HbA1c abnormality is associated with poorer thrombolytic therapy response.^21^ In contrast to our and above studies, there is few research that rejects such relationship, including the research in Korea. In this study, 639 stroke patients were evaluated. No relationship was found between HbA1c and any type of cerebrovascular lesion in the nondiabetic patients.^14^ We did not observe any relationship of HbA1c with mortality and complications in diabetic patients. This finding disagrees with some studies.^22^^,^^23^ Regardless of whether it may be an incidental finding, some factors may be involved in this discrepancy. First, the mean age of our patients was low. Second, all types of stroke were included but their frequencies were unknown. It is evident that high frequency of lacunar affects prognosis. In this study, 30.7% of patients were diagnosed with diabetes, which is within the world range. World statistics report its frequency between 15% and 44%. Such discrepancies may be attributed to some factors such as population study and definition of diabetes.^15^

**Table 3:** Comparison of mortality among diabetic and nondiabetic patients

**Time**	**Diabetic patients [n (%)]**	**Non-diabetic patients [n (%)]**	**Total [n (%)]**	**P**
30 days mortality	14 (30.4)	18 (17.3)	31 (21.3)	0.040
90 days mortality	2 (4.3)	6 (5.8)	8 (5.3)	0.721

**Table 4:** The relation between mortality and evaluated variables

**Variables**	**Crude coefficients**			**Adjusted coefficients**		
	**B**	**Adjusted odd ratio**	**P**	**B**	**Adjusted odd ratio**	**P**
Age	0.129	1.13	0.006	0.086	1.090	0.018
Gender	0	1.00	> 0.999	-0.570	0.566	0.359
HTN history	-0.077	0.46	0.302	-0.411	0.663	0.520
Smoking history	0.031	1.03	0.551	-0.230	0.794	0.709
HbA1c Hb	0.081	1.08	0.567	0.822	2.270	0.002
Hyperlipidemia history	-1.190	0.30	0.069	0.624	0.536	0.295

HTN: Hypertension; HbA1c: Hemoglobin A1c

The main limitation of this study was relatively short follow-up period. Obviously, if the follow-up period becomes longer in future studies, the results will be more valuable. In conclusion, our findings show that HbA1c can be a predictive biomarker for mortality among nondiabetic patients with AIS.

## Notes:

**How to cite this article:** Shafa MA, Ebrahimi H, Iranmanesh F, Sasaie M. Prognostic value of hemoglobin A1c in nondiabetic and diabetic patients with acute ischemic stroke. Iran J Neurol 2016; 15(4): 209-13.
